# Vesicoureteral Reflux and Other Urinary Tract Malformations in Mice Compound Heterozygous for *Pax2* and *Emx2*

**DOI:** 10.1371/journal.pone.0021529

**Published:** 2011-06-24

**Authors:** Sami K. Boualia, Yaned Gaitan, Inga Murawski, Robert Nadon, Indra R. Gupta, Maxime Bouchard

**Affiliations:** 1 Department of Biochemistry and Goodman Cancer Centre, McGill University, Montreal, Quebec, Canada; 2 Department of Pediatrics and Department of Human Genetics, McGill University, Montreal, Quebec, Canada; 3 McGill University and Genome Quebec Innovation Centre, McGill University, Montreal, Quebec, Canada; The National Institute of Diabetes and Digestive and Kidney Diseases, United States of America

## Abstract

Congenital anomalies of the kidney and urinary tract (CAKUT) are the most common cause of chronic kidney disease in children. This disease group includes a spectrum of urinary tract defects including vesicoureteral reflux, duplex kidneys and other developmental defects that can be found alone or in combination. To identify new regulators of CAKUT, we tested the genetic cooperativity between several key regulators of urogenital system development in mice. We found a high incidence of urinary tract anomalies in *Pax2;Emx2* compound heterozygous mice that are not found in single heterozygous mice. *Pax2^+/−^;Emx2^+/−^* mice harbor duplex systems associated with urinary tract obstruction, bifid ureter and a high penetrance of vesicoureteral reflux. Remarkably, most compound heterozygous mice refluxed at low intravesical pressure. Early analysis of *Pax2^+/−^;Emx2^+/−^* embryos point to ureter budding defects as the primary cause of urinary tract anomalies. We additionally establish Pax2 as a direct regulator of *Emx2* expression in the Wolffian duct. Together, these results identify a haploinsufficient genetic combination resulting in CAKUT-like phenotype, including a high sensitivity to vesicoureteral reflux. As both genes are located on human chromosome 10q, which is lost in a proportion of VUR patients, these findings may help understand VUR and CAKUT in humans.

## Introduction

Congenital anomalies of the kidney and urinary tract (CAKUT) are characterized by a high inter- and intra-familial variability in phenotypic outcome [1]. They include defects such as duplex systems, kidney and ureter agenesis, hydroureter, hydronephrosis and vesicoureteral reflux (VUR) [2]. These conditions are often associated with chronic renal disease in children. Phenotype analysis performed in mouse models with CAKUT have revealed that these defects arise at specific steps of urinary tract morphogenesis.

In the mouse, kidney and urinary tract development is initiated with the formation of the ureteric bud, a diverticulum of the Wolffian (nephric) duct. The ureteric bud invades the adjacent metanephric mesenchyme and undergoes several rounds of branching morphogenesis to form the collecting duct system of the mature kidney [1]. Ureter budding is tightly regulated such that a single kidney unit forms on each Wolffian duct. The position of the ureteric bud along the Wolffian duct is also critical to insure an appropriate insertion of the ureter in the bladder wall following ureter maturation [2]. A rostral ureter budding will typically result in vesico-ureteral obstruction (leading to the accumulation of urine in the ureter and kidney [i.e. hydroureter and hydronephrosis]), while a caudal budding site would typically result in a lateral ureter insertion site and a refluxing uretero-vesical junction [2], [3].

Crucial regulators of urogenital development have been associated with CAKUT-like phenotypes both in mouse and human [3]. Among them is the transcription factor Pax2. In the mouse, homozygous gene inactivation of *Pax2* leads to renal agenesis and other developmental anomalies, while heterozygous mice show kidney hypoplasia and VUR on an outbred genetic background [4], [5]. These defects mirror the renal hypoplasia and VUR phenotypes of Renal-Coloboma syndrome patients, resulting from mutations in the *PAX2* gene [6]. Inactivation of *Emx2* in the mouse arrests kidney development following ureter budding and invasion of the metanephric mesenchyme [7]. Other gene mutations result in CAKUT phenotypes in human and mouse [3], [8], [9]. However, few or them includes VUR as a major phenotype. Hence, despite the high frequency of VUR in humans, the causal genes remain elusive.

A number of studies have attempted to identify VUR genes by whole genome analysis of affected individuals. The two largest studies to date both identified putative regions of linkage on chromosomes 6q and 10q [10], [11]. Of notice, the distal region of chromosome 10 contains important regulators of kidney development, including *PAX2* and *EMX2*.

As the genetic cause underlying CAKUT may result from the combination of haploinsufficient loci, we assessed the genetic cooperativity between five critical regulators of urinary tract morphogenesis, namely *Pax2*, *Emx2*, *Lim1*, *Evi1* and *Gata3* [5], [7], [12], [13], [14], [15], [16], [17], [18]. These experiments revealed a strong genetic interaction between *Pax2* and *Emx2*. Compound heterozygous *Pax2;Emx2* embryos harbored a range of ureter budding defects leading to duplex systems and a very high incidence of VUR in newborn mice. We further identified a direct role for Pax2 in *Emx2* gene regulation through an enhancer located in the 3′ region of the gene. Together these results identify a regulatory cascade between two critical regulators of urinary tract morphogenesis and suggest a genetic model underlying VUR and CAKUT.

## Results

### A screen for genetic cooperativity between urinary tract developmental regulators identifies a link between *Pax2* and *Emx2*

To investigate the genetic cooperativity between essential transcriptional regulators in genitourinary tract development, we generated compound heterozygous combinations for *Pax2*, *Emx2*, *Lim1*, *Evi1* and *Gata3*, and assessed these mice for gross urogenital system anomalies at day 18.5 of development (E18.5) (Table 1). All mouse strains were bred on a pure C3H/HeJ genetic background to minimize genetic variability. Double heterozygous combinations were inspected for kidney dysplasia, duplex system and hydroureter/hydronephrosis, and the kidney surface area was measured to identify kidney hypoplasia. This analysis confirmed hypoplasia of *Pax2^+/−^* kidneys [5] and identified a novel genetic interaction between *Pax2* and *Emx2* (Table 1). None of the other allelic combinations revealed significant number of embryos with gross urogenital malformations (Table 1; data not shown).

**Table 1:** Gross anomalies in mice compound heterozygous for kidney and urinary tract developmental regulators at E18.5.

Allelic combination	wt	Gata3	Lim1	Evi1	Emx2
Pax2	1/65*	0/11	0/12	0/10	11/29#
Emx2	0/52	0/8	0/9	0/12	
Evi1	0/39	0/9	0/10		
Lim1	0/38	11-Jan			
Gata3	1/40*				

Numbers refer to embryos of the indicated allelic combination analyzed.

*The single affected Pax2^+/−^ embryo displayed a unilateral duplex kidney with duplicated collecting duct system, the Gata3^+/−^ embryo displayed unilateral hydronephrosis with associated megaureter.

#The Pax2^+/−^ Emx2^+/−^ anomalies are detailed in the text.

**Figure 1:**
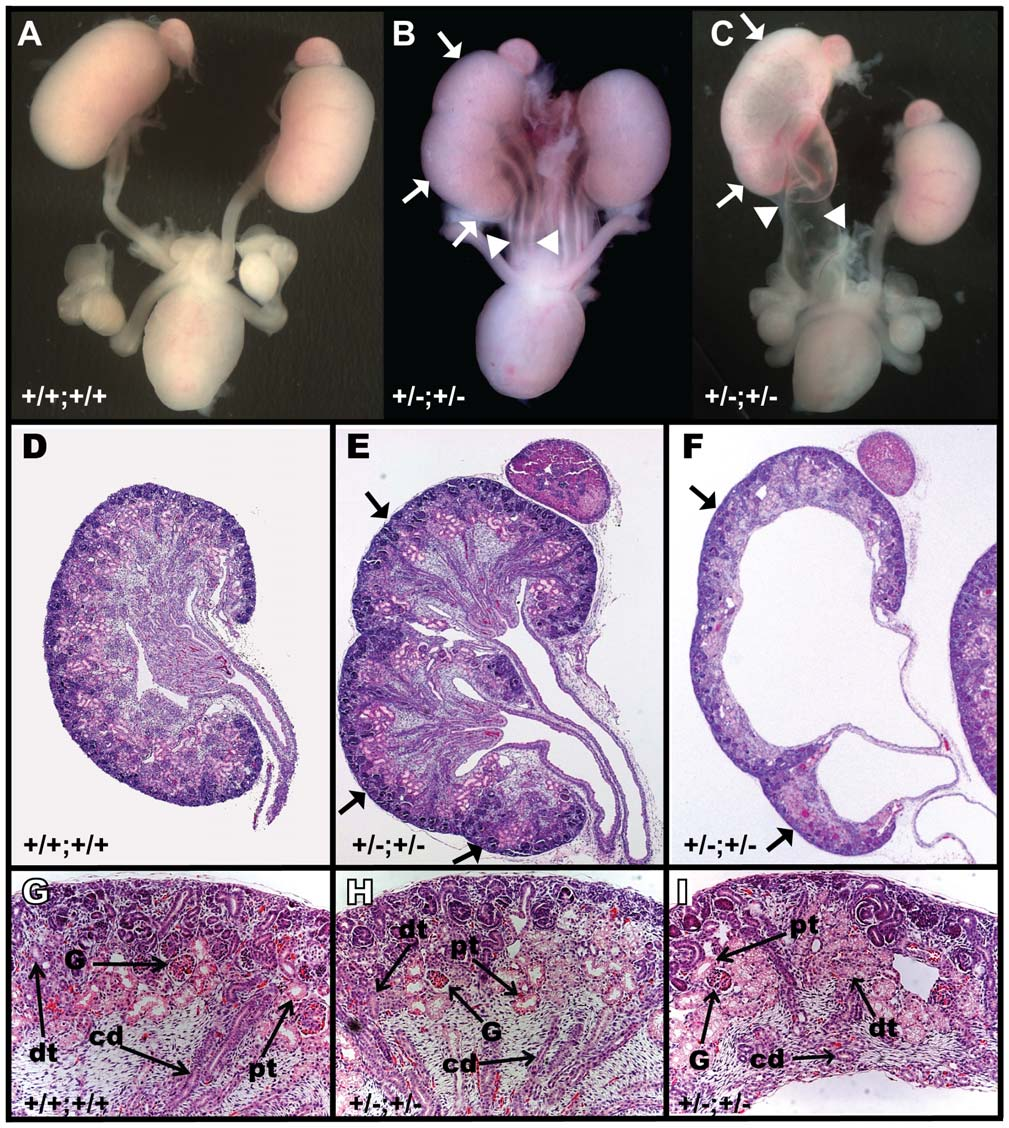
*Pax2;Emx2* compound heterozygotes display gross UGS morphological defects. (A,B,C) Isolated E18.5 urogenital systems of the indicated genotypes. (D,E,F) Hematoxylin/eosin staining of cross sections of kidneys in A, B and C, respectively. (G,H,I) Higher magnification of kidneys shown in D, E and F. Defects observed are triplex kidneys with duplex and bifid ureters (white arrows and arrowheads in B and E), and duplex kidney associated with hydronephrosis and megaureter (arrows and arrowheads in C,F). Histological analysis in G,H,I reveals normal tissue differentiation in compound heterozygotes (H,I) compared to wild-type (G). pt: proximal tubule; dt: distal tubule; G glomerulus; cd: collecting duct.

Of the *Pax2^+/−^;Emx2^+/−^* embryos inspected visually, 38% (11/29) displayed severe urinary tract malformations. These included triplex kidneys (N = 2) (including a bifid ureter) (Fig. 1B,E) and duplex systems with or without megaureter and hydronephrosis (N = 5)(Fig. 1C,F). The other malformations were milder, consisting of a single abnormally bifurcated ureter (N = 4) (data not shown). No gender bias was observed in these samples. Histological analysis failed to reveal any significant nephron differentiation defect in *Pax2^+/−^;Emx2^+/−^* kidneys (Fig. 1G,H,I). Together, these observations uncover a genetic cooperativity between *Pax2* and *Emx2* in urinary tract development.

### Severe ureter and kidney developmental defects in *Pax2^+/−^;Emx2^+/−^* embryos

**Figure 2:**
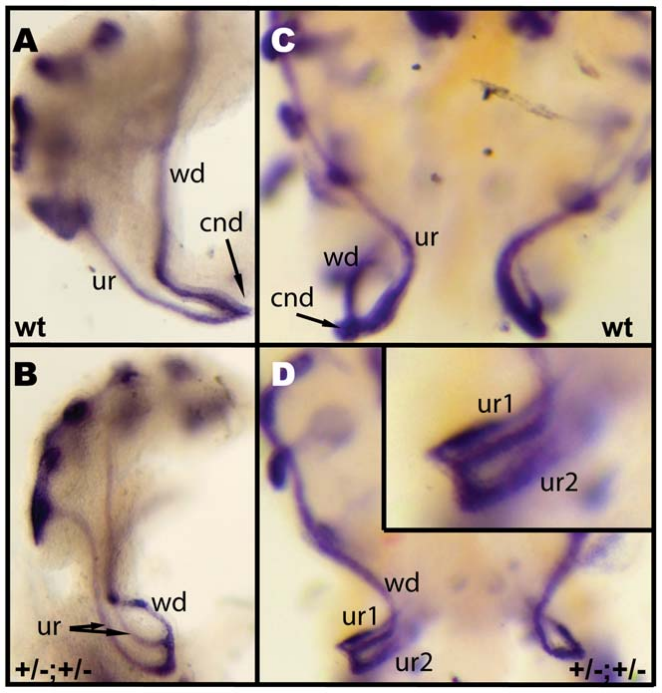
Ectopic ureter budding in *Pax2^+/−^;Emx2^+/−^* embryos. (A–D) Whole-mount *in situ* hybridization with a *Gata3* cRNA probe at E12.5. (A) Side view of wild-type (wt) urogenital system. (B) Compound heterozygote showing two independent ureters emerging from the caudal region of the Wolffian duct. (C) Ventral view of wild-type urogenital systems. (D) *Pax2;Emx2* compound heterozygote showing bifid ureteric buds pointing in opposite directions. wd: Wolffian duct, ur: ureter, cnd: common nephric duct.

The consistency of collecting duct duplication in *Pax2^+/−^;Emx2^+/−^* embryos pointed to a defect in ureter formation. To visualize the process of early urinary tract patterning, we performed whole-mount *in situ* hybridization with a *Gata3* cRNA probe at E12.5. At this stage, the ureter is undergoing maturation and joins the caudal region of the Wolffian duct, leaving a short common nephric duct (cnd) segment (Fig. 2 A,C). In *Pax2^+/−^;Emx2^+/−^* embryos, we observed duplex systems in which one ureter branched relatively high on the Wolffian duct, while the second ureter was connected with the caudal-most Wolffian duct resulting in the absence of common nephric duct (Fig. 2B). Other *Pax2^+/−^;Emx2^+/−^* embryos harbored two ureters connected with the caudal-most Wolffian duct and pointing in different directions (Fig. 2D). These results identify ectopic ureter defects in *Pax2^+/−^;Emx2^+/−^* embryos.

### High incidence of vesicoureteral reflux in *Pax2^+/−^;Emx2^+/−^* newborn

To determine whether *Pax2^+/−^;Emx2^+/−^* embryos without duplex systems had additional urinary tract defects, we initially measured the diameter of the ureter lumen of E18.5 urinary tracts. These results revealed a tendency toward ureter dilation in *Pax2^+/−^;Emx2^+/−^* (18/29 embryos) in comparison to wild-type, *Pax2^+/−^* and *Emx2^+/−^* embryos (Fig. S1A–C). As not all double heterozygous ureters were affected, these measurements did not reach statistical significance. Nonetheless, these results prompted us to further investigate ureter defects in non-duplex *Pax2^+/−^;Emx2^+/−^* embryos. For this, we first investigated ureter epithelial and mesenchymal differentiation by immunostaining against pan-uroplakins and smooth muscle actin (SMAA), respectively. These markers were found to be normally expressed in both normal and dilated *Pax2^+/−^;Emx2^+/−^* ureters, arguing against cellular differentiation defects (Fig. S1D–F).

**Figure 3:**
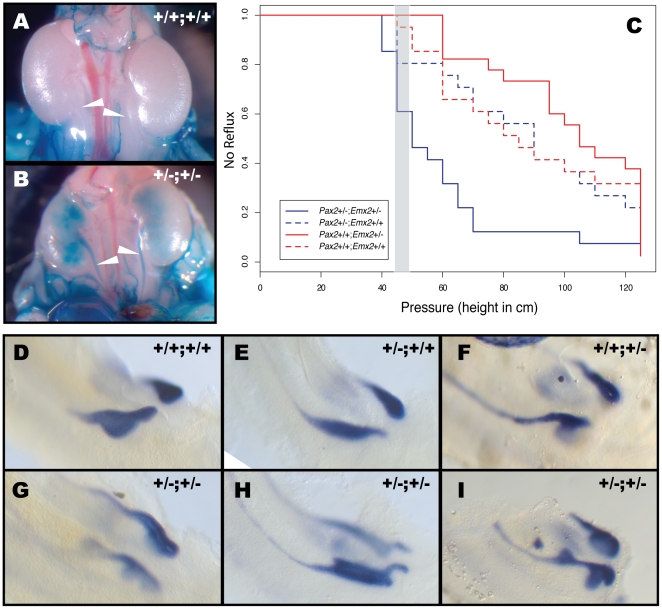
Highly penetrant VUR associated with ureter budding defects in *Pax2^+/−^;Emx2^+/−^* embryos. (A,B) Intravesical dye injection in wild-type (A) and *Pax2^+/−^;Emx2^+/−^* embryos (B) at P0. C) Graph of non-refluxing animals relative to pressure (reflected by height of dye reservoir from bladder level) for wild-type (n = 20), *Pax2^+/−^* (n = 20), *Emx2^+/−^* (n = 22) and *Pax2^+/−^;Emx2^+/−^* (n = 20). Grey area represents the average height/pressure at which the urethra voids +/− 0.5 SD. (D–I) Whole-mount *in situ* hybridization for *Ret* at E11.0 in wild-type (D), *Pax2^+/−^* (E), *Emx2^+/−^* (F) and *Pax2^+/−^;Emx2^+/−^* embryos (G–I). Note the ureteric bud malformations in compound heterozygous animals, including double buds in the same or opposite orientations, ureteric bud enlargement and ectopic projection.

We next investigated vesicoureteral reflux (VUR), as human patients with CAKUT often show distention of the ureter caused by the retrograde flow of urine towards the kidney. VUR was assessed by estimating the pressure at which dye injection into the bladder of newborn mice results in retrograde flow within the ureters [4], [9], [19]. This method is based on the linear relationship between height and pressure and consists of inserting a needle in the bladder and gradually raising the source of dye above bladder level. Strikingly, *Pax2^+/−^;Emx2^+/−^* mice were found to be much more sensitive to VUR than controls (Fig. 3A–C). At 50 cm in height, 55% of *Pax2^+/−^;Emx2^+/−^* newborns had already undergone VUR, while only 20% of controls refluxed at this pressure (Fig. 3C). Importantly, the dye consistently exited through the urethra at 45–50 cm (shaded area in Fig. 3C), which is therefore an equivalent to voiding pressure. At 70 cm in height, 90% of *Pax2^+/−^;Emx2^+/−^* newborns had undergone VUR, while only 35% of controls had (Fig. 3C). This difference identifies a highly significant interaction between the two genes (Cox Proportional Hazards analysis; p = 0.005). Hence, loss of *Pax2* and *Emx2* affect urinary tract morphogenesis, resulting in high susceptiblity to VUR.

### VUR phenotype in *Pax2^+/−^;Emx2^+/−^* mice caused by severe defects in ureter budding

VUR has been associated with an abnormal budding of the ureter in the caudal region of the Wolffian duct. In order to visualize the process of ureter budding initiation, whole-mount *in situ* hybridization was performed on *Pax2^+/−^;Emx2^+/−^* and control embryos at E10.75–E11.0 using the ureteric bud marker *Ret*. Wild-type as well as *Pax2^+/−^* and *Emx2^+/−^* embryos formed a swelling of the Wolffian duct that resolved in a well-defined ureteric bud and a clearly demarcated common nephric duct between the bud and the cloaca (Fig. 3D–F). In contrast, *Pax2^+/−^;Emx2^+/−^* embryos harbored defects in ureteric bud resolution whereby the budding field remained wider than control embryos at a similar stage and formed ectopic projections generally located in the caudal region (Fig. 3G,H, data not shown). As expected, some of these extended budding fields resolved in double/multiple ureteric buds (Fig. 3G,I). These malformations were associated with an absence of well-demarcated common nephric duct (6/6).

**Figure 4:**
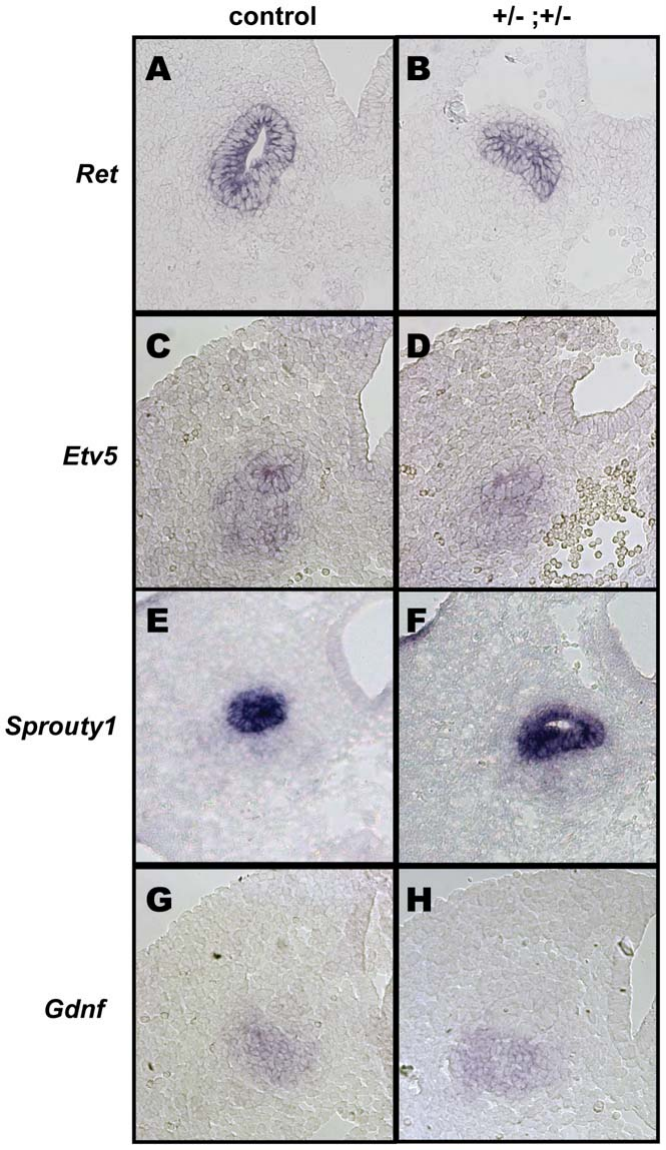
Unaltered expression of ureter budding regulators in *Pax2^+/−^;Emx2^+/−^* embryos. *In situ* hybridization for the main regulators of budding at E10.5 reveals no significant difference in mRNA expression between controls (A,C,E,G) and *Pax2;Emx2* compound heterozygous embryos (B,D,F,H). (A,B) *Ret*, (C,D) *Etv5*, (E,F) *Sprouty1,* (G,H) *Gdnf*.

To examine the molecular consequences of reduced *Pax2*/*Emx2* gene dosage on ureter budding, we performed *in situ* hybridization for components and regulators of the Gdnf-Ret pathway. We tested both epithelial markers (i.e. *Ret*, *Etv5*, *Sprouty1*), coexpressed with *Pax2* and *Emx2* in the Wolffian duct epithelium, as well as mesenchymal markers (*Gdnf*, *FoxC2*, *Bmp4*, *Gremlin*). These experiments failed to reveal any significant difference in marker expression between wild-type and double heterozygous embryos (Fig. 4 and data not shown). From these results, we conclude that the compound reduction in *Pax2* and *Emx2* expression severely affects ureter budding, through subtle or yet unidentified molecular mechanisms.

### *Pax2* is a direct transcriptional regulator of *Emx2* in the Wolffian duct

**Figure 5:**
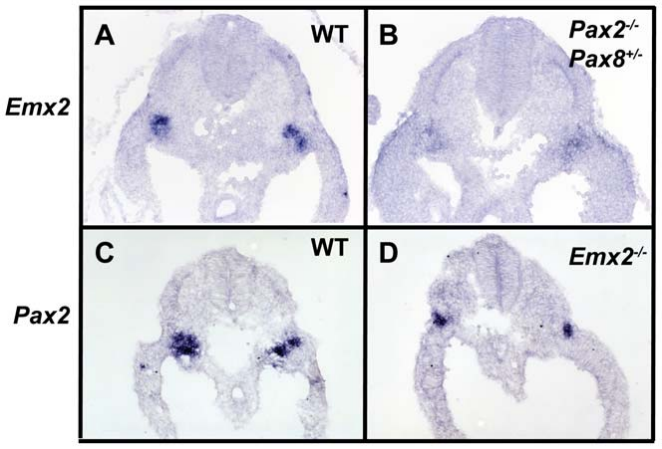
Genetic regulation of *Emx2* by *Pax2.* (A–D) *In situ* hybridization on E9.5 sections for *Emx2* (A,B), *Pax2* (C,D) in wild-type (A,C), *Pax2^-/-^;Pax8^+/-^* (B) and *Emx2^+/-^* (D) embryos. *Emx2* mRNA expression is significantly decreased in *Pax2^-/-^;Pax8^+/-^* embryos, while *Pax2* mRNA expression remains unchanged in *Emx2^+/-^* embryos.

To further characterize the genetic interaction between *Pax2* and *Emx2*, *in situ* hybridization was first performed for *Emx2* on E9.5 embryos deficient for *Pax2* and *Pax8* (*Pax2^-/-^;Pax8^+/-^*). This allelic combination was previously shown to affect *Pax* gene dosage while still allowing the formation of a pro/mesonephros [12], [14]. The expression of *Emx2* in the Wolffian duct was greatly reduced in *Pax2^-/-^;Pax8^+/-^* embryos, indicating that *Pax2/8* genes are necessary for *Emx2* expression in this tissue (Fig. 5A,B). In contrast, *Pax2* expression remained unchanged in *Emx2^-/-^* embryos (Fig. 5C,D). In these experiments *Pax8* mRNA expression was used to unequivocally identify the mesonephros on these sections (data not shown). These results indicate that *Emx2* requires *Pax* genes for normal expression in the Wolffian duct.

**Figure 6:**
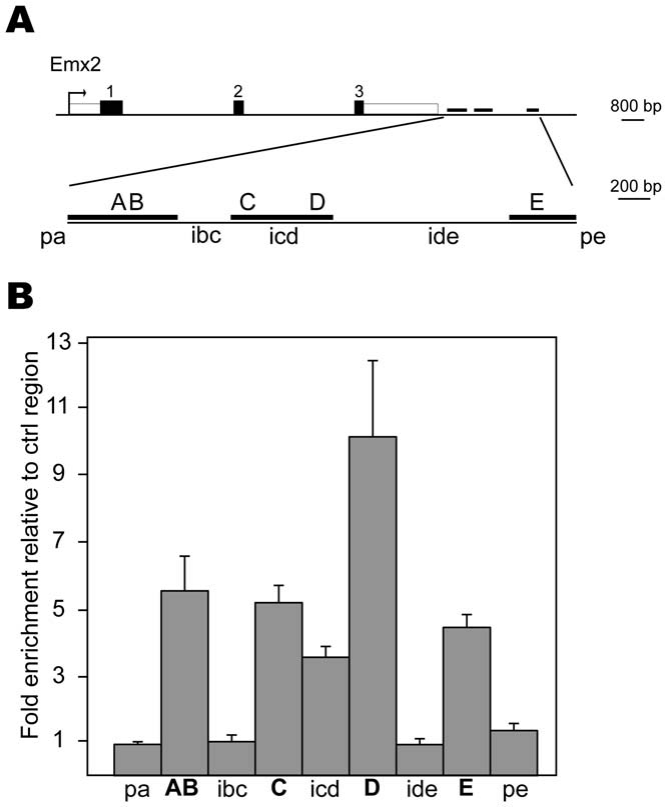
Pax2 directly binds the *Emx2* gene through a conserved 3′ regulatory region. (A) Schematic representation of the *Emx2* locus. Arrow indicates start site, black boxes exons 1,2,3 and white box indicate 3′UTR. Thick black lines represent conserved regions between mouse and human sequences. Five sites (A,B,C,D and E) were identified by bioinformatics analysis. Primer pairs used for chromatin immunoprecipitation experiment are labeled A to E and pa (pre-site A), ibc, icd, ide (inter-BC, inter-CD and inter-DE, respectively) and pe (post-site E). (B) Chromatin immunoprecipitation with anti-Pax2 antibody in mIMCD3 cells for the sites indicated in (A). Results are expressed as fold enrichment compared to an unrelated control region (near *FoxO6* gene).

To determine whether the regulation of *Emx2* by *Pax2* was direct, we initially performed a bioinformatics analysis to identify regions conserved within 200 kb of mouse and human *Emx2* sequence data (150 kb upstream and 50 kb downstream of the transcriptional start site) and then searched for Pax2 conserved consensus binding sites within these regions. We identified several putative sites, notably in five conserved segments; two upstream and three immediately 3′ of the transcribed region (Fig. 6A; Fig. S2, data not shown). We next validated all 5 putative sites by chromatin immunoprecipitation assay coupled with quantitative PCR detection (ChIP-qPCR) in mIMCD3 cells expressing endogenous Pax2. Quantitative PCR analysis on ChIP material successfully validated a cluster of five Pax2 binding sites (denoted A to E), located in the three conserved regions downstream of *Emx2* (Fig. 6B) while the other two putative sites (denoted 1 and 2) in the upstream conserved segments were not found to be enriched (data not shown). The enrichment ratios varied from 4-fold to more than 10-fold compared to controls (beads alone). To eliminate the possibility of spill-over effect between these signals, we tested regions upstream of site A (pre-A; pa), intersites (ibc, icd, ide) and downstream of site E (post-E; pe). These experiments revealed an independent binding of Pax2 on sites A/B, C, D and E. The icd probe indicated a certain level of interference between the sites C and D (separated by only 543 bp), while sites A and B were too close to test for spill-over effect (separated by 9 bp). Together these results point to a direct regulation of *Emx2* expression by Pax2, through multiple binding sites located in 3′ of the gene.

## Discussion

Congenital anomalies of the kidney and urinary tract (CAKUT), encompass a range of urogenital anomalies of variable severity. In a screen for genetic cooperativity between known regulators of urinary tract development, we found a novel mouse model that is highly sensitive to VUR and recapitulates several features of the phenotypic spectrum of CAKUT. We determined that the malformations of *Pax2^+/-^;Emx2^+/-^* embryos were caused by caudal and ectopic ureteric bud projections giving rise to additional and misplaced urinary tracts. We further ascertained that *Pax2* and *Emx2* are part of the same genetic cascade whereby Pax2 directly regulates *Emx2* gene expression in the Wolffian duct.

The cooperativity between *Pax2* and *Emx2* described here is intriguing in that *Pax2;Emx2* compound heterozygotes have a unique phenotype that is stronger and more variable than one would expect from the single heterozygote phenotypes. *Pax2^+/-^* embryos show consistent hypoplasia and have been reported to be more sensitive to reflux [4], [5]. In the present experiments, we did not see a significant difference in VUR between *Pax2^+/-^* and wild-type controls. This possibly reflects the fact that we used a genetic background (C3H/HeJ) that is already sensitive to VUR [19], masking the effect of *Pax2* haploinsufficiency for VUR. However, upon removal of an allele of *Emx2* in a *Pax2* heterozygous background, urinary tract malformations were strongly increased and now included duplex and bifid ureters as well as a very high sensitivity to VUR. This effect on VUR can be measured by the fact that reflux occurred at a pressure equivalent to voiding pressure. To our knowledge, such sensitivity to VUR has not been reported to date in other mouse models.

The double heterozygous phenotype is also more complex than the one reported for *Emx2^-/-^* embryos, which form a single ureteric bud that fails to branch following mesenchymal invasion [7], whereas no phenotype was reported for *Emx2^+/-^* embryos. As Pax2 directly regulates *Emx2*, one could expect the dosage of *Emx2* to sink below heterozygous levels in *Pax2^+/-^;Emx2^+/-^* Wolffian ducts, and therefore approach *Emx2* insufficiency levels. Instead, we observe a complex misregulation of ureteric bud induction leading to caudal ureter budding but also ectopic rostral buds and peculiar ventral projections near the caudal end of the Wolffian duct. Hence, the haploinsufficiency observed in *Pax2^+/-^;Emx2^+/-^* embryos seems to result from anomalies downstream of both transcription factors, as opposed to a simple Pax2-Emx2 linear cascade. Accordingly, the molecular defects downstream of *Pax2* and *Emx2* have proven difficult to identify, which suggests either an accumulation of subtle changes or the misregulation of yet unidentified molecular players involved in ureteric bud formation. The identity of these downstream molecular effectors and whether they primarily affect the epithelial (cell-autonomous) or mesenchymal (non-cell autonomous) compartment will be the focus of future investigations.

One possibility to explain the variability in *Pax2^+/-^;Emx2^+/-^* ureter budding anomalies is that the process of bud induction is still active but ureteric bud resolution is defective, resulting in a wider budding region that stochastically resolves in one or two ureters. Alternatively, lower gene dosage affects negative regulators of ureter budding. In either case, the primary ureter is positioned too far caudally, as reflected by the absence of clearly demarcated common nephric ducts in *Pax2^+/-^;Emx2^+/-^* embryos at E10.75–E11.0. As a result, close to 100% of *Pax2^+/-^;Emx2^+/-^* ureters are refluxing at birth. These results are compatible with the Mackie-Stephens hypothesis [20] that associates caudal ureter budding with a lateral and refluxing ureter following ureter maturation. It is also compatible with more recent models of distal ureter maturation that are based on the Mackie-Stephens hypothesis [2], [21]. Although caudal ureter budding is sufficient to explain the VUR phenotype, we cannot exclude a later distal ureter maturation problem that could further contribute to the incorrect positioning of the ureter in the bladder wall.

It is interesting to note that *PAX2* and *EMX2* are both located on chromosome arm 10q in human, namely 10q24 and 10q26, respectively. Monozygosity of 10q has been associated with a number of urogenital anomalies including VUR [22]. In addition, candidate regions for VUR were identified around chromosomal region 10q26 in two independent studies using large cohorts [10], [11]. Our results suggest that CAKUT and VUR in human may be caused by compound heterozygote combinations, which may complicate the interpretation of some of the genome-wide studies looking for the genetic determinants of these diseases. Familial cases of VUR have been associated with inheritance patterns as diverse as autosomal dominant, autosomal recessive, sex-linked and multigenic [23]. In this respect, VUR is increasingly considered as a complex trait in which a combination of genetic factors contributes to the final outcome in a single individual [23]. It will be interesting to see whether CAKUT and VUR patients are found with mutations in both *PAX2* and *EMX2*.

## Materials and Methods

### Ethics Statement

This study was approved by the Animal Care Committee of McGill University and strictly follows the guidelines from the Canadian Council on Animal Care.

### Mice

*Pax2*, *Gata3*, *Lim1*, *Evi1*, *Emx2* mice were bred on a C3H/HeJ background for at least 6 generations. Genotyping of these mice has been described previously [7], [14], [15], [18], [24]. *Evi1* mice were obtained from the Jackson Laboratory (Bar Harbor, USA).

### Ureter diameter measurements

Ureter diameter was measured from pictures taken on an Zeiss SEMI-200-C with an AxioCam MRC at a 2.5x zoom. The AxioVision-4.2 measurement tool was used to determine ureter diameter taking the caudal end of the kidney as a reference point.

### In situ Hybridization and Histology

Urogenital systems (at E11.0 and E12.5) and whole embryos at E9.5–11.0 were dissected in cold PBS and were subsequently fixed overnight in 4% paraformaldehyde at 4°C. *In situ* hybridization on E10.5 embryo cryosections with digoxigenin-dUTP RNA probes was performed as described previously [25], using the following probes: *Emx2* [26], *Ret* [27], *Gdnf* [28], *Wnt11* [29], *Slit2* [30], *Spry1* [31], *Pax2* and *Pax8* [12]. *Etv5* probe was generated from E18.5 kidney cDNA with the following primer pair 5′-TAGCAGTCCTCATCCAGGCAAC-3′ and 5′-GCAGCATCTTCCAAAGTAGGCAC-3′. *In situ* hybridization stainings at E10.5 were evaluated on metanephric mesenchyme sections at the ureteric bud level, as well as from more rostral and caudal regions. Whole-mount E11.0 and E12.5 *in situ* hybridizations using *Gata3* [32], and *Ret* [27] probes were performed as described previously [33].

### Immunofluorescence

Metanephric kidneys were dissected in cold PBS and fixed for 2 h in 4% paraformaldehyde at 4°C. Samples were processed for paraffin embedding, sectioning and subsequent immunohistochemistry as described previously [34].

### Dye injection

Vesicoureteral reflux was assessed by methylene blue injection into the bladder. Briefy, newborn mice were sacrificed and dissected, exposing the bladder, kidneys and urinary tracts. A 30-gauge needle was attached via tubing to a 60 ml syringe filled with methylene blue. After introducing the needle into the bladder, the level was raised at the rate of 5 cm/sec. from 30 to 120 cm. The rate of injection was determined by the hydrostatic pressure exerted by the weight of the column of methylene blue [9].

### Chromatin immunoprecipitaton

Murine inner medullary collecting duct cells (mIMCD3) (kindly provided by Dr. Paul Goodyer, McGill University) were cultured in a 1∶1 mix of DMEM and HAM's F12 media (Wisent) supplemented with 10% fetal bovine serum (Wisent) in all experiments. Confluent mIMCD3 cells stably expressing Gata3 (mIMCD3-Gata3) [13] were cross-linked with 1% (w/v) formaldehyde for 10 min at room temperature with mild rocking. The plates were then rinsed twice with cold PBS to stop the cross-linking reaction. The cells were collected and sonicated to achieve DNA shearing to an average of 200 bp. The chromatin was then pre-cleared with protein G-Agarose beads (Roche, cat.11243233001). The chromatin fractions were precipitated overnight with an anti-Pax2 antibody (Covance PRB-276P, LN#143834801), or beads alone as a negative control. The antibody was retrieved with protein G-Agarose beads for 2 hours, the beads were washed extensively with: low salt buffer (0.1% SDS, 1% Triton-X, 20 mM Tris-HCl (pH8), 150 mM Nacl), high salt buffer (1% Triton-X, 20 mM Tris-HCl (pH8), 2 mM EDTA, 500 mM NaCl), fresh LiCl buffer (250 mM LiCl, 1% Igepal, 1 mM EDTA, 1% Na-Desoxycholate, 10 mM Tris-HCl (pH8)) and 1x Tris-EDTA, and de-crosslinked at 65°C overnight (1% SDS, 0.1 M NaHCO_3_). The samples were then treated with proteinase K (0.2 mg/ml) for 1 hour at 55°C. The chromatin was isolated using the QIAquick PCR purification kit (Qiagen cat.28106). Quantitative PCR was performed on Pax2-precipitated samples and controls. The primers used were: Fwd-1: ATACAACCCAAGCCCTCGGATCAG-3′, Rev-1: 5′-TCCTGAGGACTTCCAGAAGACTTG-3′, Fwd-2: 5′-CCGTGTGTGTTTTCTGTTTAGCAC-3′, Rev-2: 5′-TGCTGAAAGATAGGGGAGAGTCAG-3′, Fwd-pa: 5′- CCACAAAAGAGCCAGACTGGTG -3′, Rev-pa: 5′- AAAGTTGCTTGGACAGCTTCTCTC -3′ , Fwd-AB: 5′- TGAGGGAGATGAACCCAAAGG -3′: Rev -AB: 5′- CTCGAACAGAACAGACGAGGTTTC -3′ Fwd-ibc: 5′- TGTTTTTCCTCCCCTCCTCTAAAG -3′ Rev-ibc: 5′- CAAAGTATGAGCAGCCAGGTCTG -3′ , Fwd -C: 5′- AAGTACGAGAAAGGGGAGTGGTG -3′, Rev-C: 5′- TCTTCCTACCATTGTGGGACCC -3′, Fwd-icd: 5′- GCCCAAAGAAGGATTTGATAGCC -3′ Rev-icd: 5′- TGACTGTCCTGCCTTTTTGAGG -3′ Fwd-D: 5′- AGGACAGTCAGCTTATTAGCCGC -3′ Rev-D: 5′- TACGGAACACGAGTGGGAACTC -3′ , Fwd-ide: 5′- TTGGGAAACTTGGGCTCCTC -3′, Rev-ide: 5′- TTACCACCACAGAACAGGGCAC -3′, Fwd-E: 5′- GTTTTGAGAACCCTCTGACCCC -3′ Rev-E: 5′- TGCTGGGCAACAGGCTCTAATG -3′, Fwd-pe: 5′- CGCCAAAGATCACAACCACG -3′, Rev-pe: 5′- ACCAGGAAGGAACAAAAGGGGG -3′. The results are normalized to an unrelated control region near the *FoxO6* gene on chromosome 1: Fwd: 5′- AAACCTAAGTGCTTTCTCCCTTCC -3′, and Rev: 5′- GGCTTTATCTGGTGAACAGTGGAC -3′. Putative Pax2 binding sites were identified on MacVector 8.0 program using consensus sequence (A/G)N(A/C/T)CANT(C/G)A(A/T)GCGT(A/G)(A/T)(A/C) with three mismatches allowed. This consensus was derived from validated Pax2/5/8 binding sites reported in the literature.

## Supporting Information

Figure S1 ***Pax2;Emx2*** **compound heterozygotes have enlarged ureters associated with normal smooth muscle and urothelium differentiation.** (A) Wild-type kidney and ureter at E18.5. (B) Ureter enlargement in *Pax2^+/−^Emx2^+/−^* embryos. Red bar denotes ureter diameter measurement at the level of the caudal kidney end. (C) Measurements of ureter diameter in the indicated genotypes. Horizontal bars represent averages. Urogenital systems with duplex system or megaureters have been excluded. (D) Immunofluorescent staining of ureters with smooth-muscle alpha-actin (SMAA; red) and Uroplakin (green). *Pax2^+/−^;Emx2^+/−^* embryos show dilated ureters compared to controls. No difference is seen in smooth muscle and urothelium differentiation.
(TIF)

Figure S2 **Alignment of conserved regions and Pax2/5/8 binding sites in** ***Emx2*** **3′ region.** (A) Clustal alignment of mouse and human sequences from the conserved 3′ regions. Numbers refer to start and end points of the mouse sequence (Ensembl release 58). Conserved Pax2/5/8 binding sites are bolded and shaded in gray. Up to 3 mismatches from consensus were allowed. (B) Alignment of Pax2/5/8 binding sites from *Emx2* conserved regions 1 to 3. Strand used for alignment to consensus is indicated.
(DOC)
