# Supplementary figures and images for: Vesicoureteral Reflux and Other Urinary Tract Malformations in Mice Compound Heterozygous for Pax2 and Emx2
Source: PLoS One. 2011 Jun 24;6(6):e21529. doi: 10.1371/journal.pone.0021529 (PMC3123351; doi:10.1371/journal.pone.0021529)

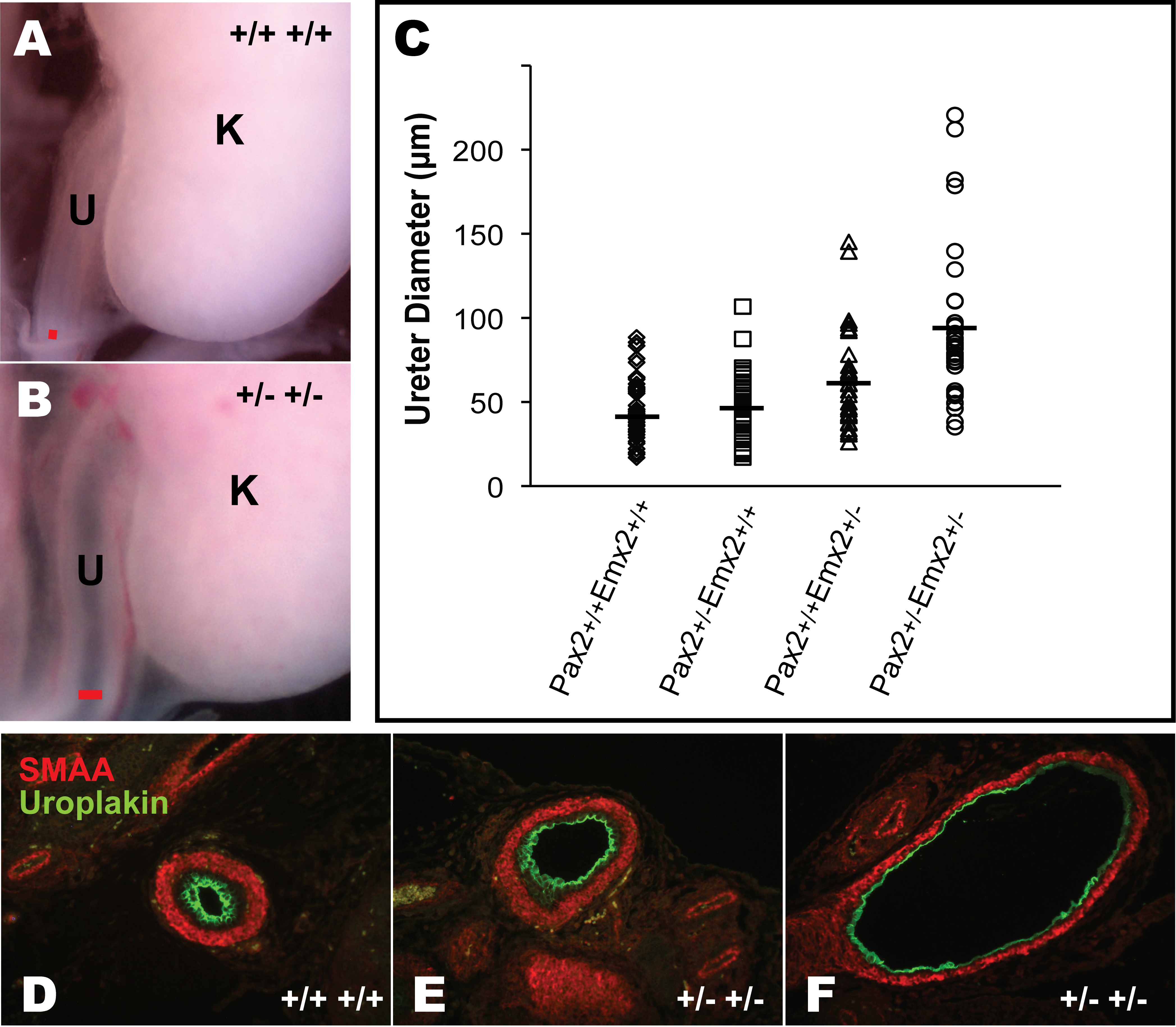

Supplement: Figure S1 — Pax2;Emx2 compound heterozygotes have enlarged ureters associated with normal smooth muscle and urothelium differentiation. (A) Wild-type kidney and ureter at E18.5. (B) Ureter enlargement in Pax2+/−Emx2+/− embryos. Red bar denotes ureter diameter measurement at the level of the caudal kidney end. (C) Measurements of ureter diameter in the indicated genotypes. Horizontal bars represent averages. Urogenital systems with duplex system or megaureters have been excluded. (D) Immunofluorescent staining of ureters with smooth-muscle alpha-actin (SMAA; red) and Uroplakin (green). Pax2+/−;Emx2+/− embryos show dilated ureters compared to controls. No difference is seen in smooth muscle and urothelium differentiation. (TIF) [file pone.0021529.s001.tif]
