# Supplementary material for: Vesicoureteral Reflux and Other Urinary Tract Malformations in Mice Compound Heterozygous for Pax2 and Emx2
Source: PLoS One. 2011 Jun 24;6(6):e21529. doi: 10.1371/journal.pone.0021529 (PMC3123351; doi:10.1371/journal.pone.0021529)
Supplement: Figure S2 — Alignment of conserved regions and Pax2/5/8 binding sites in Emx2 3′ region. (A) Clustal alignment of mouse and human sequences from the conserved 3′ regions. Numbers refer to start and end points of the mouse sequence (Ensembl release 58). Conserved Pax2/5/8 binding sites are bolded and shaded in gray. Up to 3 mismatches from consensus were allowed. (B) Alignment of Pax2/5/8 binding sites from Emx2 conserved regions 1 to 3. Strand used for alignment to consensus is indicated. (DOC) [file pone.0021529.s002.doc]

**A**

**Emx2- Region 1**

Mus musculus (Mouse genome Ensembl release 58, May 2010; NCBIM37)

Homo sapiens (Human genome Ensembl release 58, May 2010; GRCh37)

59540249-

TTTCTGACTTCAAACCCCTTCAGGACAAGTCAGCCTCCTCTTCTCTCTTTTCTTTTCTCC

TTTCTGACTTCAAACCCCA-AAGTACAAGTC---TTACTTTTCCTTCCTTTCTTTCCTCC

****************** ** ******* * ** *** ** ******* ****

TTTCCTCTCTCTTTCACTCTAATCTGCATAAAGTGAGGGAGATGAACCCAAAGGCCCCCG

TCTCCC-------TGGCCCCAATCTGCTGAAGGTGATGG-GCCAAGCCCAAGGGCCTCAG

* *** * * * ******* ** **** ** * * ***** **** * * AAGCCTTATAAGGCAAGCATTCTGAGAGATCTTCCACTCTTAATTTTAACATTAAAGTAA CAGCCTTATAAGGCAAGTGTTCTGAAAAACCTTCAACTCTTAATTTTAACATTAAAGAAA

**************** ****** * * **** ********************** ** AAGTTGTAACCATTCTCAAAGAAAACTTCGCTCTTTCCCTGTCCTCC----CCCTGCCGA AAGTTGTAATCAGACTTGGAGGAAACTTAGCTTTTTTCCCTCCCTCCTTCCCCCTTCTGG

********* ** ** ** ****** *** *** ** ***** **** * *

**(A) (B)**

GGGTATGAGGTTGTTTTT**TGCATGCTTCATTTGCT**TCCTATCCC**GATAGGCTGCATTAAT**

GGGTACGAGGTTG-TTTT**TGCATGCTTCATTTGCT**TCTTATCCT**GATAGGCTGCATTAAT**

***** ******* *********************** ***** **************** **C**AGTTTTATTTCCATTGATAGAGAAACCTCGTCTGTTCTGTTCGAGCTACAAAGCGTCCT **C**AGTTTTATTTCCATTGATAGAGAAACCTCGTCTGTTCTGTTCGAGCTACAAAGCGTCCT ************************************************************ GTGAGCTTTTGTGAAAGTGCAAATCAGTTTAAGCAATTATCATACCAGGAATATGAAGGG GCGAGCTTTTGTGAAAGTGCAAATCAGTTTAAGCAATTATCATACCAGGAATATGAAGGG

* ********************************************************** AAAAGAGGAGGCCTTGCCCAGTGGTCTCCTTTAATCTCTTAATCCACGGATCCAGGGGGG GAAAGAGGAGGCCTTGCCCAGTGGTCTCCTTTAATCTCTTAATCCACGGATCCAGGGGGG

***********************************************************

CTGCCTGGACATAATTTAGGACAATCTCCCCCACCCTTTACACTGTGATAAGGCCAAGTT

CTGCCTGGACATAATTTAGGACAATCTCCCC-ACCCTTTACACTGTGATAAGGCCAAGTT

******************************* ****************************

ACAATGCAGGGGCAAAAATACAAGCTTTGTTAACATCCTGCCTTGAAAAGTTAAGATTAG

ACAATGCAGGG-CAAAAATACAAGCTTTGTTAACATCTTGCCTTGAAAAGTTAAGATTAG

*********** ************************* ********************** ACATTCCGTGCACGTGTGGGGACTTTAGGGCACTATGGAATCTTTTCTTTCTGTTTTTCC ACATTCCGTGCACGTGTGGGGACTATAGGGCACTATGGAAATTTTT---TCTTTTTTTTC

************************ *************** **** *** ***** *

TCCCCTCCTCTAAAGGAGAATTCATTCTC -59540933

TCCTCTTCTCTAAAGCAGAATTCATTCTC

*** ** ******** *************

**Emx2- Region 2**

Mus musculus (Mouse genome Ensembl release 58, May 2010; NCBIM37)

Homo sapiens (Human genome Ensembl release 58, May 2010; GRCh37)

59541378-

TTTAAGTACGAGAAAGGGGAG----TGGTGAAGAGTTTTTTTTCT------AAAT**GTCCT**

TTTAAGTACAAGAAAACGGGGCTGGTGGTGAAGATTTTTCTTTTTTTGGGTAAAT**GTCCT**

********* ***** ** * ********* **** *** * *********

**(C)**

**ATGCAGTGTGAC**AGCAATAAAATCATCAGAGAATACTATTAGCTTTGAGGGGGAAAAAAA

**ATGTAGTGTAAC**AGCAATAAAATCATCACAGAATACTATTAGCTTTGA----------AA

*** ***** ****************** ******************* **

AAAAACCTAAAAGCTTTATTACAAACCAAGCTTTGAAAATAGGGGGGATTAGGCGGCTGA

AAAAAACTAAAAGCTTTATTACAAACCAAGCTTTGAAAATAGGGGGGATTAGGCGGCTGA

***** ******************************************************

AAGGGTCCCACAATGGTAGGAAGAAAAGGTTTCATGCTAATGAGGTTAATGCCCTTTGTA

AAGGGTCCCACAATGGTAAGAAGAAAAGGTTTCATGCTAATGAGGTTAATGCCCTTTGTA

****************** *****************************************

TCTCGGGCCTCCACATCTTCATTACGCGCTATCTCCGGCTGCACCGAGCGGCTCAGAGAG

TCTCAGGCCTCCACATCTTCATTACGCGCTATCTCCGGCTGCACGGAGCGGCTCAGAGAG

**** *************************************** ***************

CCGCAATCCACTCCAACGCCCCCCTTCCCGGCCCAAAGAAGGATTTGATAGCCGCTCTGT

CCGCAATCCACTCCAACGCCCCCCTTCCCGGCCCAAAGAAGGATTTGATAGCAGCTCTGT

**************************************************** *******

TCAAACTAGATAATTATATCTTTTCAAGTCGGAATTAAGATAAAGAAAGTGAAGCAGAGG

TCAAACTAGATAATTATATCTTTTCAAGTCGGAATTAAGATAAAGAAAGTGAAGCAGAGG

************************************************************

CGGCTCGCCTTGATCCACTGCAAACAAATTTGGCGCACTTTCGAGTCCTTCCCCCCGCCT

CGGCTCGCCTTGATCCACTGCAAACAAATTTGGCGCACTTTCGAGTCCTT-CCTCCGCCT

************************************************** ** ******

CAAAAAGGCAGGACAGTCAGCTTATTAGCCGCTCGTTTGCTTTATTAATTCATCTATTTA

CAAAAAGGCAGGACAGTCAGCTTATTAGCCGCTCGTTTGCTTTATTAATTCATCTATTTA

************************************************************

AAGTGGCAGGATTAGAGCGTCTAATGTGGGCGCGGGCTGCCGTGGCGCTGAAGAATATAA

AAGTGGCAGGATTAGAGTGTCTAATGTGGACGCGGGCTGCCGTGGCGCTGAGGAATATAA

***************** *********** ********************* ********

**(D)**

ATATTTGCGAGATGCC**ATCCCACGATGCCTATC**TG -59542002

ATATTTGCGAGATGCC**ATCCCACGATGACTATC**TG

*************************** *******

**Emx2- Region 3**

Mus musculus (Mouse genome Ensembl release 58, May 2010; NCBIM37)

Homo sapiens (Human genome Ensembl release 58, May 2010; GRCh37)

59543633-

CCCCAACCAGCCCCGTAGCGAGGGGGAAGTTGGAGTTTTGAGAACCCTCTGACCCCAGCC

CCCCAGCCAGCCCCGTAGCCAGAGGAAAGTGGGAGTTTTGAGAACCCTCTGACCCCAGCC

***** ************* ** ** **** *****************************

GTGACCCGGCTCCGGGAGGCAACTTCTCCAGGCGCGATCCTGTGCGCCAGGAAACCCGAA

GTGACCCCTCTCCGGGAGGTGGCTTCTCCC-GCAGGACCCGGTGCGCCAGAAAACTCTAG

******* ********** ******* ** ** ** ********* **** * *

**(E)**

GCAGT**GCCACGCTTGAAAGCGA**AAGGTCCATTCATCAGCCGCGCCCCAGCCTCTAATTGG

GCGGC**GCCACGCTTGGAAGCGC**AAGGTCCATTCATCAGCCGCGCCCCAGCGTCTAATTGG

** * ********** ***** **************************** *********

CCTTTTGTTCATTAGAGCCTGTTGCCCAGCAGGGGAGCTTGGGAGGG-CCCGAGGCTGCA

CCTTTTGTTTATTAGTGTCTGTTGCCCGGCTGGGAGTCTCCGGAGGGCCCCGGAGCTGCG

********* ***** * ********* ** *** ** ****** **** *****

AGTCGGGGTGCCTGCTGCAGGCTGGGGACCGGTGCCTGTGCCCGGGGCTGAGGTCTCTAT

AGTCGGGGTGCCAGAAGCGGACGGCGG------GCCAGCGCCCAGAGCTGGGGTCCGGGT

************ * ** * * * ** *** * **** * **** **** *

AAGGGCAAAAGC-CAACGCGCTCCTCTGAGGGGGCGCCAGAGCGCGCCAAAGATCACAAC

-AGGGCAGAGGCGCGTCGCACTCCTCTGAGGGGGCGCCAGAGCGCGCCAAAGATCACGCC

****** * ** * *** ************************************* *

CACGTGCAAAAATGCGCTCCTGGAGGCTAGTCCCCAAGAGTCAGGGCCTAGTCCT- 59544045

CACGTGCGGAGATGCGCGCATGGAGGCTGGACTCCAAGTGCTGGAGCCTGGTCCT

******* * ****** * ******** * * ***** * * **** *****

**B**

**Site A-** AGCAAATGAAGCATGCA (minus strand)

**Site B-** GATTAATGCAGCCTATC (minus strand)

**Site C-** GTCACACTGCATAGGAC (minus strand)

**Site D-** ATCCCACGATGCCTATC (plus strand)

**Site E-** TCGCTTTCAAGCGTGGC (minus strand)

**Consensus** GNCCANTCAAGCGTAAA

A A G T GTC

T
